# Relationship Between Gastrointestinal Symptoms in Autism Spectrum Disorder and Parent Stress, Anxiety, Depression, Quality of Life and Social Support

**DOI:** 10.1007/s10803-023-06110-7

**Published:** 2023-09-01

**Authors:** Arlene Mannion, Geraldine Leader

**Affiliations:** Irish Centre for Autism and Neurodevelopmental Research, School of Psychology, University of Galway, Galway, Ireland

**Keywords:** Autism spectrum disorder, Gastrointestinal symptoms, Comorbidity, Parent stress, Parental quality of life

## Abstract

Gastrointestinal (GI) symptoms are a common comorbidity in children and adolescents with autism spectrum disorder (ASD). Little is known about the impact that GI symptoms have on parental well-being. Parents of 409 children and adolescents with ASD completed the GI Symptoms Inventory, Parenting Stress Index-Short Form, World Health Organization Quality of Life Abbreviated Version, Hospital Anxiety and Depression Scale, and the Multidimensional Scale of Perceived Social Support. High levels of stress were demonstrated by parents with 40.1% receiving clinically significant scores. A relationship was found between parental stress and GI symptoms. Parental anxiety and depression were found at high levels but were not more common in parents of individuals with GI symptoms than those without. Lower levels of quality of life were found in parents of individuals with GI symptoms compared to parents of individuals without GI symptoms. Parents of children with GI symptoms were less satisfied with their personal and social relationships with others. Parents of children with GI symptoms had lower scores on a measure of perceived social support than parents of children and adolescents without GI symptoms. GI symptoms are stressful for parents and future research is needed to determine how to alleviate this stress and to improve the quality of life of parents of individuals with ASD.

## Introduction

### Gastrointestinal Symptoms in ASD

Gastrointestinal symptoms have been recognized as a comorbid condition in individuals with autism spectrum disorder (ASD) (Holingue et al., 2023; Leader et al., 2022a, 2022b, 2022c, 2022d, 2022e). It is understood that GI symptoms such as constipation, diarrhea, abdominal pain, nausea, and bloating can cause pain and discomfort to children and adolescents with ASD (Leader & Mannion, 2016; Leader et al., 2022a, 2022b, 2022c, 2022d, 2022e). However, little is known about the effects that these symptoms have on parents and the family unit. We know that these GI symptoms can have a profound effect on a child’s well-being, yet we do not know how these symptoms affect a parent’s well-being. While research has been conducted on parental stress in parents of children with ASD (Higgins et al., 2023; Lanyi et al., 2022), little research has included GI symptoms as a potential contributing variable to parental stress, anxiety, depression, poorer quality of life, and less perceived social support.

### Relationship Between Gastrointestinal Symptoms in ASD and Parent Variables

#### Stress

GI symptoms are linked to reduced quality of life in children and adolescents with ASD, as well as sensory issues, challenging behavior, comorbid psychopathology, and sleep problems (Leader et al., 2022a, 2022b, 2022c, 2022d, 2022e). Therefore, GI symptoms exacerbate other behavioral issues and there is a link between GI symptoms and other increased symptoms. The relationship between GI symptoms and challenging behavior is one that has been explored in previous research (Leader et al., 2022a, 2022b, 2022c, 2022d, 2022e; Restrepo et al., 2020).

Similarly, the relationship between challenging behavior and parental stress has been explored in previous research (Higgins et al., 2023; McStay et al., 2014a). A relationship has been established between GI symptoms and challenging behavior. Previous research has indicated that there is a relationship between challenging behavior and parental stress (Lanyi et al., 2022). The potential relationship between GI symptoms and parental stress needs to be investigated. One could hypothesise that an increase in GI symptoms would lead to an increase in parental stress. While other researchers examined the relationship between parental stress and challenging behavior in parents of children and adolescents with ASD, often other variables such as GI symptoms, are often not considered.

Research has examined the effects of stress in parents of children with ASD (Foody et al., 2014; Iwamoto et al., 2023) and has examined the differences between fathers and mothers of children with ASD (Foody et al., 2015). While we know that higher levels of stress are prevalent in parents of children and adolescents with ASD, very little is known about the impact GI symptoms have on parental stress.

Silva and Schalock (2012) investigated stress in parents of children with autism, under the age of six. The research examined the core symptoms of autism, the comorbid behavioral symptoms, and the comorbid physical symptoms. The researchers investigated bowel problems, including constipation and diarrhea as a comorbid physical condition. Bowel problems were found to be stressful for 31.8% of parents of children with ASD, compared to 5% of parents of typically developing children and 17.9% of parents of children with other developmental disabilities. Parents of children with ASD who exhibited bowel problems differed significantly from parents of typically developing children in parenting stress levels. However, no significant differences were found between parents of children with ASD and parents of children with other developmental disabilities for bowel problems. It was found that 9.3% of parents of children with autism endorsed bowel problems as “Very stressful on a daily basis” while 5.6% of parents endorsed these problems as “So stressful sometimes we feel we can’t cope”.

Toileting training issues were also found to be a stressor for parents. Silva and Schalock (2012) found potty training to be stressful for 50.5% of parents of children with autism, compared to 8.6% of parents of typically developing children. It was found that 24.3% of parents of children with autism endorsed bowel problems as “Very stressful on a daily basis” while 11.2% of parents endorsed these problems as “So stressful sometimes we feel we can’t cope”.

#### Anxiety and Depression

Anxiety and depression are important areas of study in parents of children and adolescents with ASD (McIntyre et al., 2023; Roubinov et al., 2023). Foody et al. (2015) reported that mothers of children with ASD reported higher levels of parental distress, anxiety, and depression than fathers. This is supported by Ozturk et al. (2014) who found that mothers of children with ASD reported higher levels of depression than fathers of children with ASD. Ozturk et al. (2014) found a relationship between parental distress and parental depression. Pozo and Sarriá (2015) investigated stress, anxiety, depression, and well-being in three groups; parents of adults with ASD, parents of adolescents with ASD, and parents of young children with ASD. It was found that depression and anxiety were lower in parents of adults and adolescents with ASD, compared to parents of young children with ASD.

Falk et al. (2014) investigated parental predictors of stress, anxiety, and depression in parents of children with ASD. Anxiety was predicted by maternal age, and the mother’s perceived ability to set behavioral limits. Depression was predicted by child’s aggressive behavior, a perceived lack of social support, and an externalized parental locus of control. Kuhlthau et al. (2014) found that 40% of parents of children with ASD reported having clinical depression symptoms. The authors found that married parents reported lower depression symptoms than parents who were not married. Kuhlthau et al. (2014) also found that parents who had two or more children with special health care needs had more clinical depression symptoms than parents who had one child with special health care needs. It was found that 51% of parents of children with ASD reported moderate or severe problems with anxiety or depression. Foody et al. (2014) found that 19% of parents of children with ASD had anxiety scores in the severe range. The authors found that increased number of unmet service needs predicted higher maternal depression.

#### Quality of Life

Recent research has focused on quality of life in parents of children with ASD (Adams et al., 2020; Davy et al., 2023). Kuhlthau et al. (2014) investigated health-related quality of life in parents of children with ASD. Health; especially stress and mental health, of parents of children with ASD was found to be lower than normative populations. Focus groups were also conducted and it was found that parental health-related quality of life was negatively influenced by their child’s ASD. Parents mentioned that stress was caused by the child’s behavioral problems. In parents of children with ASD, McStay et al. (2014c) found that child behavior problems were associated with father’s perceptions of family quality of life. The authors commented that more parents may stay at home due to their child behavior problems, and “their satisfaction with their quality of life may decrease due to being unable to actively engage in the community” (McStay et al., 2014c, p. 3111).

McStay et al. (2014b) examined maternal stress and quality of life in parents of children with autism from preschool age to adolescence. It was reported that 17.6% of mothers of preschool children, 41% of mothers of early school age children, 17.9% of mothers of middle school age children, and 37% of mothers of early high school age children reported high satisfaction with their family quality of life. It was found that high levels of satisfaction fluctuated between the different age groups. Mothers of early school age children and adolescents reported significantly higher levels of satisfaction with their family quality of life than mothers of preschool and middle school age children.

#### Social Support

Research has been conducted on the relationship between perceived social support and a number of variables, including posttraumatic growth (Feng et al., 2022), subjective well-being (Bi et al., 2022), parental stress (McGrew & Keyes, 2014), anxiety and depression, and quality of life (Hsiao, 2016). Very little is known about the possible relationship between perceived parental social support and whether a child presents with GI symptoms or not. It may be hypothesised that parents of children with GI symptoms may be less likely to seek access to supportive opportunities, such as in-person parent groups. Parents may also be less likely to engage in leisure and community participation activities which could be a supportive opportunity for them. Certain GI symptoms may be very difficult for parents, such as when a child is experiencing abdominal pain or other physical symptoms such as diarrhea and constipation. These GI symptoms can limit access to supportive opportunities for parents. This may lead parents to avoid unnecessary journeys outside the child’s home and school environments, and therefore a parent may have less access to social support. This hypothesis is supported by Davy et al. (2023) who indicated that there are barriers to participation in meaningful activities for parents of children with ASD. However, as so little is known about GI symptoms and perceived social support, the current study aims to explore this relationship further.

#### Current Study

The current study aims to explore the relationship between child GI symptoms and parental well-being. The relationship between GI symptoms and parental stress, anxiety, depression, quality of life, and social support will be examined. The aim of the study is to provide data on how GI symptoms affect the well-being of parents of children and adolescents with ASD.

## Method

### Participants

Participants were 409 parents of children and adolescents with a diagnosis of ASD in accordance with DSM-IV-TR criteria (American Psychiatric Association, 2000) Diagnoses were provided by a licensed psychologist or pediatrician independent of the study. The participants received their diagnosis as a result of the formal diagnostic protocol which employs multiple diagnostic measures. Caregiver information on professional diagnosis, diagnostic setting/organization and professional(s) who made the diagnosis was obtained. Of the parents, 95.4% (*n* = 390) were female while 4.6% (*n* = 19) were male. The mean age was 40.34 years (*SD* = 6.82), while the age range was from 24 to 62 years.

### Parental Measures

#### Parenting Stress Index, Fourth Edition Short Form (PSI-4-SF)

The Parenting Stress Index-Short Form (PSI-4-SF; Abidin, 2012) identifies parent–child problem areas in parents. It contains 36 items, and is divided into three domains; Parenting Distress (PD), Parent–Child Dysfunction Interaction (P-CDI), and Difficult Child (DC). These domains combine to form a Total Stress score. It is rated on a five-point Likert scale from 1(Strongly Agree) to 5 (Strongly Disagree). Coefficient alphas for each PSI-4-SF are all above 0.90. The psychometric characteristics of the PSI-4-SF have been examined (Reitman et al., 2002).

#### Hospital Anxiety and Depression Scale (HADS)

The Hospital Anxiety and Depression Scale (HADS; Zigmond & Snaith, 1983) is used to assess anxiety and depression. It contains 14 items, and has two subscales; Anxiety and Depression. Responses are rated on a 4-point Likert scale ranging from 0 (Not at all) to 3 (Very Often Indeed). A score of 0–7 is classified as normal for each score, 8–10 is classified as borderline abnormal (borderline case) and 11–21 is classified as abnormal. Bjelland et al. (2002) reviewed the validity of the HADS. Cronbach’s alpha was found to be 0.68 to 0.93 (mean = 0.83) for the Anxiety subscale, while it was from 0.67 to 0.90 (mean = 0.82) for the Depression subscale. Correlation between the HADS and other commonly used questionnaires were in the range 0.49 to 0.83.

#### World Health Organization’s Quality of Life Questionnaire-BREF (WHOQOL-BREF)

The World Health Organization’s Quality of Life Questionnaire-BREF (WHOQOL-BREF; The WHOQOL Group, 1998) was used to assess parent quality of life. It is a shorter version of the original instrument, the WHOQOL-100. It contains 26 items and has a number of different domains including physical health, psychological health, social relationships, and environment. Items are scored on a five-point scale. The WHOQOL Group (1998) found that the WHOQOL-BREF correlated highly with the domains of the original WHOQOL-100. WHOQOL-BREF domain scores demonstrated good discriminant validity, content validity, internal consistency, and test–retest reliability. The WHOQOL-BREF has been used with parents of children with intellectual disabilities (Lin et al., 2009). It has recently been validated for use with parents of children with ASD (Dardas & Ahmad, 2014).

#### Multidimensional Scale of Perceived Social Support

The Multidimensional Scale of Perceived Social Support (MSPSS; Zimet et al., 1988) was used to assess perceived social support. It contains 12 items. Each item is rated on a 7-point Likert scale from 1(Very Strongly Disagree) to 7 (Very Strongly Agree). The items are divided into factor groups relating to the source of social support, which are Family, Friends or Significant Other. High internal consistency has been demonstrated (Canty-Mitchell & Zimet, 2000). Good internal reliability and strong factorial validity has been demonstrated (Zimet et al., 1990).

### Child Measures

#### Gastrointestinal Symptom Inventory

The Gastrointestinal Symptom Inventory (Autism Treatment Network, 2005) is a 35-item questionnaire that was developed in the early days of the Autism Treatment Network (ATN). There are also additional items should a participant exhibit certain symptomatology, and therefore includes 77 items in total. The ATN is the first network of hospitals and physicians dedicated to developing a model of comprehensive medical care for children and adolescents with autism through seventeen participating institutions in the U.S. and Canada. It was based on previous questionnaires and on clinical symptom assessment for children with autism and identified gastrointestinal disorders. The inventory is scored initially dichotomously i.e. whether or not the child has any gastrointestinal symptoms. The inventory also allows branching into specific areas of symptomatology: abdominal pain, abnormal bowel movements, reflux, and food insensitivity. These branches will allow determination of rates of these categories as well. This tool has not been validated. However, a brief version of the scale has been developed, the GI Signs and Symptoms Inventory (GISSI-17; Margolis et al., 2019) and was used in published research (Bresciani et al., 2023). The original Gastrointestinal Symptom Inventory has been used in a number of published research studies (Leader et al., 2020; Leader et al., 2021a; Leader et al., 2022a, 2022b, 2022c, 2022d, 2022e; Leader et al., 2022d; Leader et al., 2022e; Leader et al., 2021b; Mazefsky et al., 2014; Mazurek et al., 2013).

### Procedure

Parents and guardians were made aware of the study through schools, ASD service providers, parent support groups and online forums. If parents wished to participate in the study, they were provided with a participant information sheet and a consent form to complete. Once consent was obtained, parents were provided with the battery of the above questionnaires to complete in their own time.

## Results

### Analyses

Pearson correlations were conducted to determine if there were associations between variables. Chi-Square tests were used to determine associations between nominal variables and whether parents of children and adolescents had GI symptoms or not. *T*-tests or MANOVAs were conducted to assess differences of parents of children and adolescents who display GI symptoms compared to those with no GI symptoms. Bonferroni adjustment for multiple comparisons was applied.

### Demographic Information

Demographic information is included in Table 1. An association was found between parent gender and child GI symptoms, X^2^ (1, *N* = 408) = 8.21, *p* = 0.013. It was found that 96.4% of mothers reported GI symptoms in their children, compared to 3.6% of fathers. No association was found between GI symptoms and parental level of education (*p* = 0.290), marital status (*p* = 0.216), or whether parent was currently ill (*p* = 0.421). No significant difference was found between parent age (*p* = 0.305) and whether a child had GI symptoms or not.

**Table 1:** Demographic information

Variable	*n* (%)
Age	*M* (40.34 years) *SD* (6.82 years)
Gender	
Male	19 (4.6%)
Female	390 (95.4%)
Education level	
No education	1 (0.2%)
Primary education	6 (1.5%)
Secondary education	141 (34.5%)
Tertiary education	261 (63.8%)
Marital status	
Single	29 (7.1%)
Married	281 (68.7%)
Living as married	39 (9.5%)
Separated	27 (6.6%)
Divorced	31 (7.6%)
Widowed	2 (0.5%)
Currently Ill	
Yes	76 (18.6%)
No	333 (81.4%)

### Relationship Between Child Gastrointestinal Symptoms and Parent Variables

#### Parenting Stress and Child Gastrointestinal Symptoms

On the PSI-SF, it was found that 2.2% (*n* = 9) of parents engaged in defensive responding. Defensive responding was significant if a participant scored a sum of 10 or less on a selected number of items of the PSI-SF. A summary of the PSI-SF subscale means, standard deviations, cut-offs across participants, and results of the *t*-tests are shown in Table 2. It was found that parents of children with GI symptoms scored significantly higher on the subscale Difficult Child (*m* = 40.63) than parents of children without GI symptoms (*m* = 36.93).

**Table 2:** PSI-SF means, standard deviations and cut-off scores

Subscale	*M*	*SD*	*t*	*p*	Normal		High score		Clinically significant	
					*n*	%	*n*	%	*n*	%
Parental distress (PD)	36.13	10.12	− 1.21	.226	231	56.5	23	5.6	155	37.9
Parent–child dysfunctional										
Interaction (PCD-I)	31.68	8.81	− 1.58	.116	244	59.7	30	7.3	135	33
Difficult child (DC)	40.22	9.42	− 2.52	.012*	153	37.4	34	8.3	222	54.3
Total score	107.73	24.39			211	51.6	34	8.3	164	40.1

*p < .05

#### Parent Anxiety and Depression and Child Gastrointestinal Symptoms

A summary of the HADS subscale means, standard deviations, cut-offs across participants, and results of the MANOVA are displayed in Table 3.

**Table 3:** HADS means, standard deviations, and cut-off scores

Subscale	*M*	*SD*	*F*	*p*	Normal		Borderline abnormal		Abnormal	
					*n*	%	*n*	%	*n*	%
Anxiety	9.88	4.31	.63	.427	131	32	98	24	180	44
Depression	7.76	4.12	1.90	.169	193	47.2	110	26.9	106	25.9

#### Parent Quality of Life and Child Gastrointestinal Symptoms

It was found that 22% (*n* = 88) of participants rated their quality of life as very good, while 51% (*n* = 204) rated it as good. It was found that 19% (*n* = 76) considered their quality of life neither good nor poor, while 7.5% (*n* = 30) considered it to be poor, and 0.5% (*n* = 2) considered it to be very poor. It was reported that 13.3% (*n* = 53) were very satisfied with their health, 43% (*n* = 172) were satisfied, and 18.8% (*n* = 75) were neither satisfied nor dissatisfied. It was found that 20.5% (*n* = 82) were dissatisfied and 4.5% (*n* = 18) were very dissatisfied with their health. A summary of the WHOQOL-BREF domain means, standard deviation, and results of the MANOVA can be found in Table 4. It was found that parents of children and adolescents with GI symptoms had significantly lower scores on the domain of Social Relationships in the WHOQOL-BREF (*m* = 52.68) than parents of children and adolescents without GI symptoms (*m* = 63.65). Lower scores were found in the WHOQOL-BREF domain of Environment for parents of children with GI symptoms (*m* = 62.45) than parents of children without GI symptoms (*m* = 69.57).

**Table 4:** WHOQOL-BREF means and standard deviations

Subscale	*M*	*SD*	*F*	*p*
Physical health	65.27	19.12	1.84	.176
Psychological health	60.08	19.06	.71	.339
Social relationships	53.98	24.17	8.12	.005**
Environment	63.28	17.98	6.48	.011*

*p < .05, **p < .01

#### Parent Social Support and Child Gastrointestinal Symptoms

A summary of the MSPSS means, standard deviations and results of the *t*-test are shown in Table 5. A significant difference was found between parents of children with and without GI symptoms on their perceived social support, *t* (406) = − 4.21, *p* = 0.016. Parents of children with GI symptoms had lower scores of perceived social support (*m* = 53.69), compared to parents of children without GI symptoms (*m* = 60.57). Specifically, there was a significant difference between parents of children with and without constipation on their perceived social support, *t* (374) = − 3.53, *p* = 0.000, with parents of children with constipation presented with lower scores of perceived social support (*m* = 52.20), compared to parents of children without constipation (*m* = 59.24).

**Table 5:** MSPSS mean and standard deviation

Variable	*M*	*SD*	*t*	*p*
MSPSS total score	54.46	18.35	− 2.41	.016*

*p < .05

## Discussion

Parents of children and adolescents with ASD experienced high levels of stress in general with 40.1% of parents receiving clinically significant total scores on the Parenting Stress Index-Short Form (PSI-SF). A relationship was found between parental stress and GI symptoms. Specifically, the PSI-SF subscale of Difficult Child was significantly higher in parents of children and adolescents with GI symptoms than in those without GI symptoms. A child presenting with GI symptoms led parents to report more difficulties relating to stress with their child. This is not a surprising finding, as it was hypothesised that having a child with GI symptoms would be a stressful experience for parents.

No relationship was found between anxiety and depression in parents of children and adolescents with or without GI symptoms. As can be seen, anxiety is a very common symptom among parents of children with ASD regardless of whether their child presents with GI symptoms or not. This is in line with previous research which found anxiety and depression to be very common issues among parents of children with ASD (Foody et al., 2014, 2015; Higgins et al., 2023; Lanyi et al., 2022).

Lower levels of quality of life were found in parents of children and adolescents with GI symptoms compared to parents of children and adolescents without GI symptoms. Specifically, parents of children with GI symptoms had lower scores on the WHOQOL-BREF domains of Social Relationships and Environment. Parents of children with GI symptoms are less satisfied with their personal and social relationships with others. This may be due to a parent having a large burden of care due to caring for a child who presents with GI symptoms. Parents of children with GI symptoms also experience quality of life issues in relation to their environment. Our finding of the relationship between GI symptoms and reduced parental quality of life is a novel finding. Future research needs to expand on this and is needed to better understand the role that GI symptoms play on parental quality of life.

The finding of lower quality of life in terms of social relationships is supported by our finding of lower levels of perceived social support in parents of children with GI symptoms. Parents of children with GI symptoms had lower scores on a measure of perceived social support than parents of children and adolescents without GI symptoms. Specifically, it was found that there were lower measures of perceived social support in parents of children who experienced constipation, in comparison to parents whose children did not present with constipation. This finding highlights the need for more support to be given to parents and the importance of services, such as respite care which could give parents some time to encourage social relationships, which in turn could possibly be a good support to them in the future.

It is important to consider the findings of this study in relation to treatment of GI issues. Alleviating GI issues could be a point of intervention for the child with ASD and their families. It is possible that interventions for GI problems could have an impact on the well-being of parents. That is, if parents of children with GI symptoms display higher stress levels, lower quality of life, and lower perceived social support than parents of children without GI symptoms, alleviating GI issues may have an impact on parental stress, quality of life, and perceived social support. Current research on interventions for GI conditions is promising and an avenue for future research (Leader et al., 2022a, 2022b, 2022c, 2022d, 2022e; Troisi et al., 2020).

A relationship was found between parental stress and GI symptoms. Parental anxiety and depression were found at high levels but were not more common in parents of individuals with GI symptoms than those without. Lower levels of quality of life were found in parents of individuals with GI symptoms compared to parents of individuals without GI symptoms. Parents of children with GI symptoms were less satisfied with their personal and social relationships with others. Parents of children with GI symptoms had lower scores on a measure of perceived social support than parents of children and adolescents without GI symptoms.

In summary, this study provided novel data on the relationship between child GI symptoms and parental well-being. The parenting stress of Difficult Child on the PSI-SF was found to be greater in parents of children with GI symptoms, compared to those without GI symptoms. We found that anxiety and depression are common issues in parents of children and adolescents with ASD, regardless of whether children present with GI symptoms or not. Parents of children with GI symptoms had reduced quality of life, especially in social relationships and the environment, in comparison to parents of children with no GI symptoms. A key finding was the relationship between perceived social support in parents and the presence of GI symptoms in children and adolescents. GI symptoms are affecting the amount of social support that parents are receiving. Likewise due to the other challenges associated with GI symptoms, such as challenging behavior, parents may perceive that they have less social support. GI symptoms are stressful for children with ASD and for their parents. All too little research focuses on GI symptoms and all too often parents do not receive guidance and support on GI symptoms from healthcare professionals due to the lack of knowledge in this area. By conducting more research on GI symptoms, more awareness will be given to this area of research and the most effective treatment options can be determined.
